# A Spiking Neuron Model of Word Associations for the Remote Associates Test

**DOI:** 10.3389/fpsyg.2017.00099

**Published:** 2017-02-02

**Authors:** Ivana Kajić, Jan Gosmann, Terrence C. Stewart, Thomas Wennekers, Chris Eliasmith

**Affiliations:** ^1^School of Computing, Electronics and Mathematics, University of PlymouthPlymouth, UK; ^2^Centre for Theoretical Neuroscience, University of WaterlooWaterloo, ON, Canada

**Keywords:** semantic search, vector representations, semantic spaces, Neural Engineering Framework (NEF), spiking neurons, remote associates test

## Abstract

Generating associations is important for cognitive tasks including language acquisition and creative problem solving. It remains an open question how the brain represents and processes associations. The Remote Associates Test (RAT) is a task, originally used in creativity research, that is heavily dependent on generating associations in a search for the solutions to individual RAT problems. In this work we present a model that solves the test. Compared to earlier modeling work on the RAT, our hybrid (i.e., non-developmental) model is implemented in a spiking neural network by means of the Neural Engineering Framework (NEF), demonstrating that it is possible for spiking neurons to be organized to store the employed representations and to manipulate them. In particular, the model shows that distributed representations can support sophisticated linguistic processing. The model was validated on human behavioral data including the typical length of response sequences and similarity relationships in produced responses. These data suggest two cognitive processes that are involved in solving the RAT: one process generates potential responses and a second process filters the responses.

## 1. Introduction

Language acquisition is highly dependent on the ability to create associations (Elman et al., 1997; Rogers and McClelland, 2004), as they are a central means of expanding both vocabulary and syntax (Brown and Berko, 1960; Hills, 2013). As well, associations allow infants to learn about previously unseen objects or concepts in terms of semantic similarities and semantic distinctions (Mandler and McDonough, 1993). While acquisition of language occurs in the earliest stages of human growth and development, starting with utterances of simple words and sentences, language skills continue to develop over the lifetime. Because associative mechanisms play such a crucial role in language and human cognition more generally, it is important to understand how the brain might represent, store, and deploy them.

The representation of linguistic content is an actively researched topic in various disciplines. For example, in Natural Language Processing (NLP) researchers work on optimal representations for the extraction of information from large corpora of text, as well as algorithms for text comprehension and production. Technology companies such as Facebook and Google are actively researching how to make machines better at understanding human language to improve their services and the efficiency of interactions between machines and humans (Mikolov et al., 2013; Bordes et al., 2014; Weston et al., 2014; Hermann et al., 2015). One of the primary goals in NLP is to reach high performance on practical problems. Because this goal is generally adopted without regard for psychological or biological plausibility, it is unclear how such approaches can provide useful insights into how the brain solves the same problems.

Potentially more promising contributions to understanding the representation of lexical content and its meaning in the brain come from neuroimaging studies (Binder et al., 2009). Several studies have used fMRI data to construct semantic maps spanning broad areas of the cerebral cortex (Huth et al., 2012, 2016). Also, direct brain stimulation in the frontal cortex, left perisylvian cortex, and posterior temporal cortex of epileptic patients has identified regions essential for language production and comprehension (Ojemann et al., 1989).

While such studies provide us with a high-level perspective on possible brain regions involved in the processing of language, they do not shed light on how words and word associations might be represented by individual neurons and small networks. Improving our understanding of these lower-level mechanisms is a daunting task due to difficulty of locating, accessing, and recording from the brain regions responsible for such processes. In addition, direct recordings of neurons are invasive and can seldom be done on healthy humans.

Here, we opt to use a modeling approach to circumvent these problems while still gaining insight into representational structures and mechanisms that may potentially be used by the brain. We chose a linguistic task, the Remote Associates Test (RAT), to verify that the chosen representations of words and associations allow the model to perform in correspondence with human behavioral data. The model is hybrid insofar as it does not simulate the developmental process underlying the neural behavior. Rather, we use an analytical approach to derive the neural connectivity and then use simulated spiking neurons to produce the search process in the RAT. This makes it a non-developmental neural model, and we believe this is an important step toward the ultimate goal of having a complete neural account of the entire process that results in RAT behavior.

The choice of a particular neuron model also represents an important decision in the process of constructing the model. While there is a wide variety of neural models, we have chosen the leaky integrate-and-fire (LIF) neuron model due to its favorable trade-off between computational efficiency, analytical tractability, and its ability to capture some of the basic features of neuronal dynamics observed in biological systems (Izhikevich, 2007). In particular, synaptic dynamics and noise from fluctuations introduced by spiking impose constraints that a theoretical approach used to simulate neural systems needs to account for. The LIF neuron model is a spiking neuron model as it imitates the spiking behavior observed in biological neurons.

In biological neurons, electrically charged ions are exchanged across the cell membrane and an influx of positive ions into the cell can cause the neuron to trigger an action potential (also known as a spike). A spike can be registered by another, receiving neuron, if it has a synaptic connection with the neuron emitting a spike. In our modeling approach, spiking neurons are also connected by synapses so that the arrival of a spike at the side of a receiving neuron causes a post-synaptic current. The relevant neuron and synapse model parameters such as the membrane and synaptic time constants, and the shape of the post-synaptic currents conform to empirically measured value ranges and properties. These constraints are ensuring that the modeled system approximates the biological system and provides an account of the internal mechanisms underlying the investigated behavior.

### 1.1. The remote associates test (RAT)

The RAT was developed in the early 1960s (Mednick, 1962) to study an individual's ability to think creatively. A creative thought or idea can often be described as novel and unusual (Boden, 2003). In the RAT subjects are presented with three cue words and have to find a solution word related to all cues within a time limit. An aspect of creativity is thought to be captured by subjects generating solution words that are only remotely associated with the problem cues, requiring subjects to relate familiar words in a novel way. For example, given a cue triplet *fish, mine*, and *rush*, thinking about common associations of each of the triplets such as *water, coal*, and *hour* is not helpful. Instead, *gold*, a less frequent associate of each of the words, is the correct solution as it can be meaningfully combined with each of the cues. The associative relationship between the cues and the solution in the RAT can vary: it can be a compound word such that each cue and the solution form a new word (e.g., *firefly*); it can be semantically related (e.g., *water* and *ice*); or it can form an expression (e.g., *mind game*). Mednick (1962) proposed that creative individuals are more likely to think of unstereotypical words that are solutions in the RAT. He attributed this to their flat associative hierarchy, in which the probability of coming up with an association is not very different for typical and untypical associations. In contrast, individuals scoring lower on the RAT would produce stereotypical associates with higher probability than untypical associates, which Mednick (1962) described as the characteristic of a steep associative hierarchy.

Performance on the test is expressed as the number of correctly solved items within a time limit, which is typically somewhere between a few seconds and a few minutes. Longer intervals correlate with higher solution rates (Bowden and Jung-Beeman, 2003), and it is assumed that longer solving periods allow for deliberate search processes, while shorter solving times are more likely to reflect sudden and involuntary insight solutions (Kounious and Beeman, 2014). Analyses of responses people give when attempting to solve a RAT problem have shown particular search patterns that differentiate search in the RAT from other related search processes (Raaijmakers and Shiffrin, 1981; Hills et al., 2012). Specifically, the RAT search process retrieves words that are strongly related to one of the three problem cues, shows occasional switching between the cues (Smith et al., 2013; Davelaar, 2015), and involves a local search strategy (Smith et al., 2013; Smith and Vul, 2015).

Performance on the RAT has been characterized by experimental, theoretical, and computational studies (Gupta et al., 2012; Kenett et al., 2014; Klein and Badia, 2015; Olteteanu and Falomir, 2015). Mednick's proposal about flat associative hierarchies of high-scoring individuals has been supported experimentally by studies showing that indviduals who score higher on the RAT tend to avoid high-frequency answers on both incorrect and correct trials (Gupta et al., 2012; Kenett et al., 2014). This observation was further supported using NLP approaches that achieve better-than-human performance on the RAT (Klein and Badia, 2015; Olteteanu and Falomir, 2015). The properties of individual subjects' semantic networks correlates with their performance on the RAT (Kenett et al., 2014; Monaghan et al., 2014). Specifically, individuals who score high on a battery of creativity tests have semantic networks with small-world properties (Kenett et al., 2014). The connectivity in such networks is sparse, as they are characterized by short average path lengths between words, and strong local clustering. However, even though every node in the network is only sparsely connected, it takes just a few associations to reach any other node in the network. This kind of topology would assist in the solution of the RAT because quick, efficient searches can cover much of the semantic network.

### 1.2. Neural representation

The question of word representation is central to all models concerned with linguistic tasks, including the RAT. Borrowing from the early theories of semantic memory in cognitive psychology (Collins and Quillian, 1969; Collins and Loftus, 1975), it is reasonable to approach the RAT by creating a semantic network where individual words are represented as nodes connected via edges indicating associations. Then, the process of finding the solution involves either a random or directed search in the network. Indeed, several models have used such representations to demonstrate performance on par with human performance (Bourgin et al., 2014; Monaghan et al., 2014; Kajić and Wennekers, 2015).

In terms of neurally plausible representations, these models would most closely correspond to the localist theory of representation (Bowers, 2009). Localist representations imply that a single neuron or a small group of neurons carries meaning. While this approach is often considered problematic in that it implies the existance of so-called “grandmother cells” (where there are particular neurons dedicated to representing the concept “grandmother”), some support for this type of representation can be seen in studies recording from single-cells which show high degrees of specificity in their response to external stimuli (Hubel and Wiesel, 1968; Moser et al., 2008; Quian Quiroga, 2012). In contrast to localist representations, distributed representations (McClelland and Rumelhart, 1987; Rogers and McClelland, 2004) are characterized by the assumption that a concept is represented by a population of neurons, where each individual neuron participates in the representation of multiple concepts. More recently, it has been argued that the kind of data used to support localist representations is often exhibited by distributed models (Stewart and Eliasmith, 2011; Eliasmith, 2013, p. 98–99, 369–370). Importantly, as will be described in more detail below, the method for distributed representation of concepts used in this paper suggests that each neuron within a distributed representation has a preferred state. This means that some neurons might be highly specific while others will have broad responses in our biologically informed distributed representation (Stewart et al., 2011a).

### 1.3. Modeling the remote associates test

Despite arguments and evidence that distributed representations are used in many parts of the brain, there is no agreed upon approach to characterizing the representation of cognitive or linguistic structures using such representations. In particular, it is an open question of how such representations support word associations and how they might be employed in tasks requiring associative processing. We suggest answers to these questions by building a model that bridges from individual spiking neurons to the behavioral level and validating it on the RAT task.

To construct the model, we used the Neural Engineering Framework (NEF; Eliasmith and Anderson, 2003) described in the following section. It allows us to derive the required neural network to implement the necessary representations and transformations for performing the RAT. We describe the specific model in Section 2.3 and evaluation methods in Section 2.4. The quantitative and qualitative results are presented in Section 3, followed by a discussion and concluding remarks.

## 2. Materials and methods

The hybrid model presented in this paper was constructed with the methods of the NEF (Eliasmith and Anderson, 2003). The NEF specifies how a wide variety of functions can be implemented in biological neurons. It has been successfully used to model a diverse set of neural systems including those controlling behaviors such as eye position control, directed arm movements, and lamprey locomotion (Eliasmith and Anderson, 2003). It has also been successfully applied to the modeling of higher cognitive tasks such as serial working memory and action selection, and was the basis for the construction of the first detailed brain model capable of performing multiple tasks, called Spaun (Eliasmith et al., 2012). In this section we introduce the essentials of the NEF required to represent words with neural populations and to manipulate these representations. Using these basic methods, we describe the organization of a neural network to realize the cognitive processes in RAT memory search. We conclude by describing the semantic analysis methods used to validate the model.

### 2.1. Neural engineering framework (NEF)

We first describe how a group of neurons encodes a vector-valued stimulus ***x***, which lays the foundation for the representation of single words. Neurons have preferred stimuli, that is, they will respond more strongly to some stimuli than to other stimuli. For example, neurons in the striate cortex show selective responses to vertical bars of different orientations (Hubel and Wiesel, 1968) and neurons known as place cells in the hippocampus selectively exhibit specific firing patterns when an animal is present in a particular location in an environment (Moser et al., 2008). This stimulus preference can be expressed by assigning a preferred direction vector ***e***_*i*_ to each neuron *i*. The inner product $e_{i}^{⊤}x$ expresses how strongly a neuron will respond to a given stimulus; it increases as the stimulus vector aligns with the preferred direction. This value can be thought of as being proportional to the amount of current flowing into a neuron, leading to the equation

(1) $$\begin{array}{l} a_{i}\left(t\right)=a_{i}\left(x\left(t\right)\right)=G_{i}\left[\alpha_{i}e_{i}^{⊤}x\left(t\right)+J_{i}^{bias}\right] \end{array}$$

which gives the neuron activity *a*_*i*_(*t*) at time *t* for a time-dependent stimulus ***x***(*t*). Here we convert the inner product into an input current to a neuron by means of a gain factor α_*i*_ and a bias current $J_{i}^{bias}$, used to capture observed neural responses also known as neural tuning curves. The spiking activity *a*_*i*_ of a neuron is given by applying a neuron non-linearity *G*_*i*_ to the input current.

**Figure 1:**
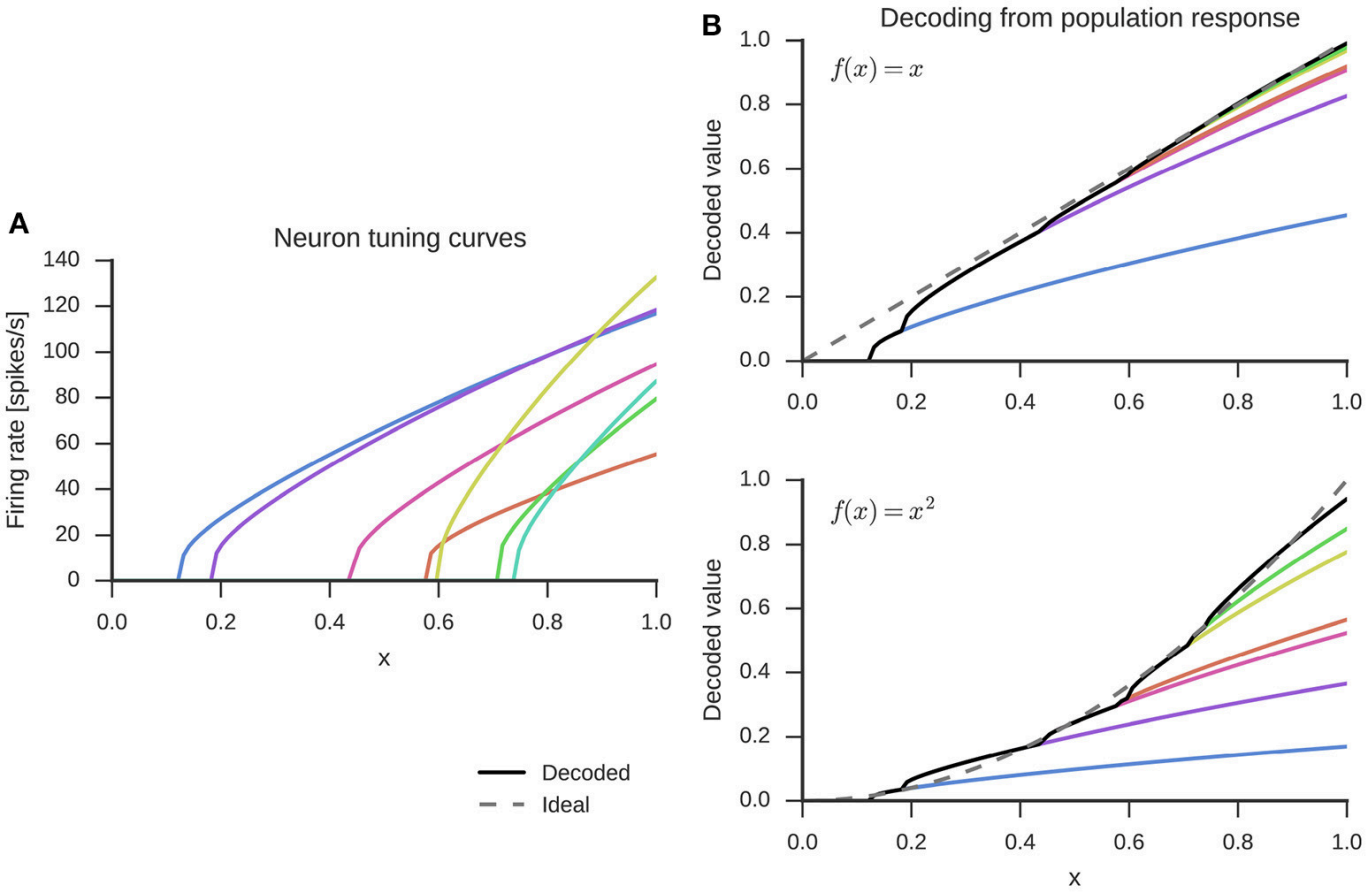
**Randomly generated tuning curves for seven neurons (left)** and linear combination of these to decode the represented value *x* **(top right)** or decode a function, here *x*^2^ **(bottom right)**. The dashed gray line is the ideal output and the black solid line the decoded value from all seven neurons.

While a wide variety of neuron non-linearities can be used with the NEF, here we use the LIF neuron model, which captures important properties related to neuronal excitability observed in biological neurons (Koch, 2004, Chapter 14). The incoming currents are accumulated as membrane voltage until a firing threshold is reached. At that point, the neuron emits a spike and the membrane voltage is reset to its resting value for a refractory period during which the neuron is unable to produce spikes. Without incoming currents, the membrane voltage will slowly decay to a resting potential due to leak currents. The left panel of Figure 1 shows an example of how individual neurons in a set of seven LIF neurons respond to inputs in the range *x* ∈ [0, 1]. In this one-dimensional space, all preferred directions are either −1 or 1. For this example specifically, we assigned preferred directions of 1 to all neurons, as indicated by the increasing firing rate with increase of *x*. This captures the effect where stronger environmental stimuli (larger values of *x*) elicit stronger neural responses.

Given the firing in a group of neurons, how do we reconstruct the represented value ***x***? With LIF neurons, *a*_*i*_(*t*) is a spike train, i.e., *a*_*i*_(*t*) is 0 at all times *t* that no spike occurred and peaks at the spike times. However, biologically, each spike causes a post-synaptic current, which can be modeled as an exponential filter of the form $h\left(t\right)=\frac{1}{\tau }\exp\left(-t/\tau \right)$. This function can be combined with a linear decoding to provide a weighted linear filter that estimates the original vector ***x***. That is:

(2) $$\begin{array}{l} \hat{x}\left(t\right)=\underset{i}{\sum }a_{i}\left(t\right)*\left[d_{i}h\left(t\right)\right]\text{.} \end{array}$$

The weights ***d***_*i*_ are obtained by a global least-squares optimization of the error $E=\underset{k}{\sum }||x_{k}-{\hat{x}}_{k}|{|}^{2}$ of the reconstructed stimulus across *k* sample points and all neurons in the group. The decoding process is visualized in the top right panel of Figure 1. The decoding weights scale the tuning curves (left panel) and the represented value is estimated with a sum over the scaled tuning curves.

Representing and reconstructing values is not sufficient for functionally interesting neural networks. Information needs to be transmitted and manipulated between groups of neurons. To do this, we need to find the synaptic connection weights that will perform this transformation. These can be computed from the decoding weights ***d***_*i*_ of the pre-synaptic neurons that reconstruct an estimate of the represented value $\hat{x}$. In addition, the input current to a post-synaptic neuron *j* depends on its preferred direction ***e***_*j*_ and gain α_*j*_. Because the quantities ***d***_*i*_, ***e***_*j*_, and α_*j*_ do not change over time^1^, they can be multiplied together to provide standard neural network connection weights as follows

(3) $$\begin{array}{l} W_{ji}=\alpha_{j}e_{j}^{⊤}d_{i} \end{array}$$

where *W*_*ji*_ comprise the synaptic weight matrix ***W***. This is the optimal synaptic connection weight matrix for transmitting information from one neural group to another (Eliasmith and Anderson, 2003).

Finally, in addition to finding synaptic weight matrices that simply pass information from one group of neurons to the next, the NEF also allows us to find weight matrices that will compute functions *f*(***x***) with neurons. This is done by using alternate decoding weights $d_{i}^{f}$. Again, these can be determined from a least-squares optimization, but with a different error function $E^{f}=\underset{k}{\sum }||f_{k}\left(x\right)-\hat{f_{k}}\left(x\right)|{|}^{2}$. The decoding with such alternative weights for the example of *f*(*x*) = *x*^2^ is shown in the bottom right panel of Figure 1. The optimization for finding decoding weights is done separately for each function, but over all neurons within a group of neurons at once.

To summarize, the NEF allows us to state how a time-varying, vector-valued stimulus is encoded in neural populations, how the value represented in a neural population can be decoded, and how to connect neural populations to compute functions using those represented values. All connection weights are determined in an offline optimization without the need for an online process.

### 2.2. Representing words and associations with the NEF

To model the word search process in the RAT, words and associations among them need to be represented. We centrally adopt a representation where the activity of several neurons contributes to a representation of multiple words. In the NEF, this is achieved by using vectors to represent words, which we have elsewhere referred to as *Semantic Pointers* (Eliasmith, 2013)^2^. With the random distribution of preferred direction vectors ***e***_*i*_, each neuron will be involved in the representation of multiple words and the representation is distributed across the neurons.

Representing words as vectors has a long tradition within NLP, for example Latent Semantic Analysis (Deerwester et al., 1990; Landauer and Dumais, 1997) and word2vec (Mikolov et al., 2013) are just two prominent approaches of generating word vectors from text corpora. Approaches like LSA and word2vec usually try to encode semantic structure or associations into the similarity or distance between the vectors. However, given two associated words A and B, this makes it difficult to decide which of these words is represented under the noisy conditions of plausible spiking neural representations. The word vector for A might become more similar to B than to A due to the noise. Thus, we avoid this kind of representation and use nearly orthogonal vectors. Specifically, we generate random unit-vectors with the constraint that no pair of such vectors exceeds a similarity of 0.1 as measured by the dot product. To fulfill the similarity constraint, a sufficient vector dimensionality has to be used. For the *N* = 5018 words used in the model, we set the dimensionality to *D* = 2048. This is considerably below the number of words because the number of almost orthogonal vectors, that can be fit into a vector space, grows exponentially with the number of dimensions (Wyner, 1967).

Such vector-based word representations have been successfully used to implement a variety of cognitive tasks such as the Tower of Hanoi task (Stewart and Eliasmith, 2011), inferential word categorization (Blouw et al., 2015), and Raven's Advanced Progressive Matrices (Rasmussen and Eliasmith, 2014). These representations have been shown in simulation to be robust to neural damage (Stewart et al., 2011a) and are consistent with the type of distributed representation found throughout sensory and motor cortex (Georgopoulos et al., 1986).

We next turn to the methods we used to compute the connection matrix between groups of neurons representing associations. These methods refer to algebraic operations and we do not consider them to be a part of the model. Instead, we use them to compute a matrix $\tilde{A}$, which is implemented in connection weights among groups of neurons. The matrix $\tilde{A}$ is used to describe associations between words and it transforms a word vector ***w*** to a linear combination of its associates. This matrix can be derived from an association matrix ***A*** where *A*_*ij*_ gives the associative strength from word *i* to word *j*. To do so we need to define the *N* × *D* matrix ***V*** that collects all the word vectors, i.e., row *i* of ***V*** is the vector representing word *i*. Then we can state $\tilde{A}=V^{⊤}A^{⊤}V$. Applied to a vector ***w***, this will first correlate *w* with all the word vectors (***Vw***) to yield an *N*-dimensional vector indicating the similarity with each word; then ***A***^⊤^ is used to retrieve the corresponding associations before ***V***^⊤^ projects those associations back into a *D*-dimensional vector. As all of this collapses into a single linear transformation matrix $\tilde{A}$, the retrieval of associations can be easily implemented with the NEF in the connection weights between two groups of neurons, computing the function $y=\tilde{A}x$.

The model assumes that this set of connection weights is given. That is, we do not aim to explain the underlying developmental process, or speculate on whether particular mechanisms are innate or acquired developmentally. We would expect that the learning of associations and word representations occurs separately from the search process. Prior work (Bekolay et al., 2013; Voelker, 2015) has demonstrated the learning of NEF connection weights with spiking neurons in a biologically plausible manner, but we leave the investigation of these processes in this context to future work.

### 2.3. Model description

We describe the core parts of the model most relevant to the RAT here, omitting implementational details not relevant to the main model function. A complete description can be found in the Supplementary Material. We used the Nengo neural network simulator (Bekolay et al., 2014) for the implementation of the model. The model source code can be found at https://github.com/ctn-archive/kajic-frontiers2016.

**Figure 2:**
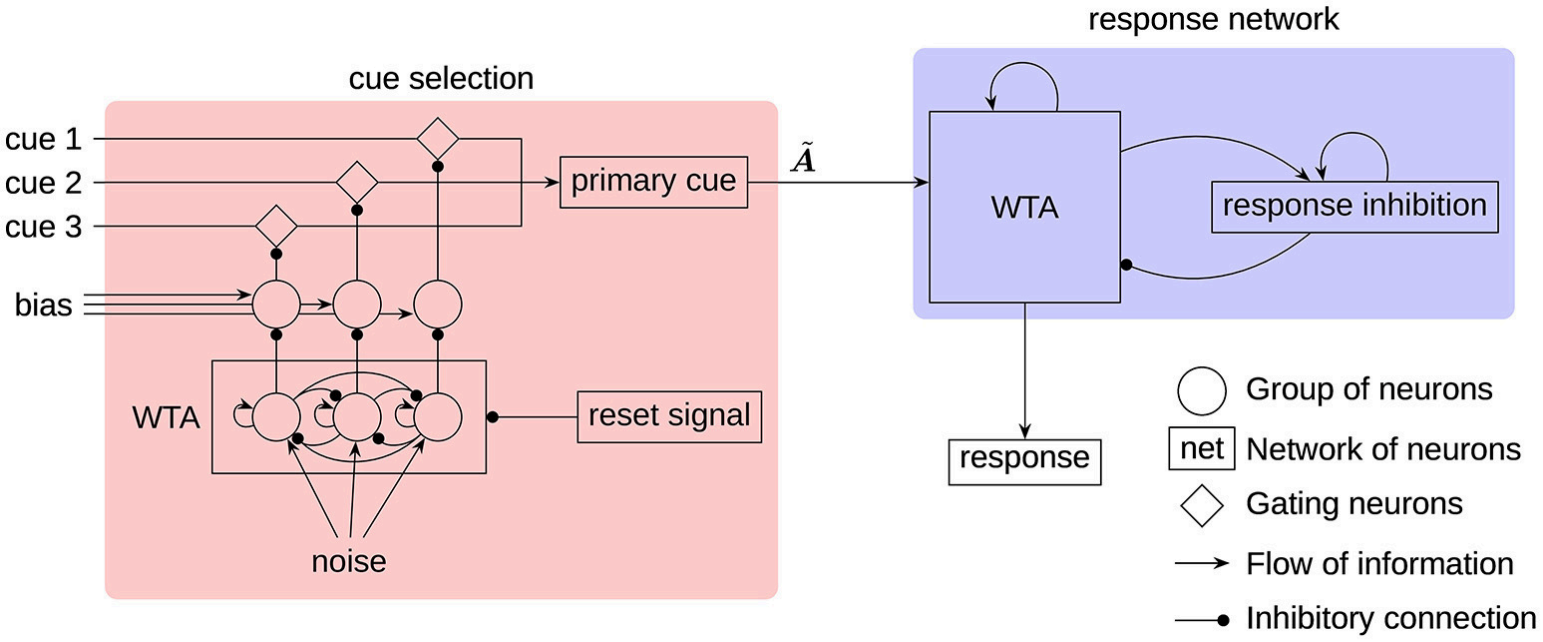
**The architecture of the RAT model**. All neural groups, gating neurons, and networks consist of spiking neurons. The cues, noise, and bias are provided as external input to the model. The $\tilde{A}$ label indicates the transformation, implemented on the connection to produce the associates of the primary cue.

All components of the model can be grouped into two main parts (see Figure 2):

- A *cue selection network* that randomly selects one of the three input cues as the primary cue. This selection is repeated in certain intervals to allow the primary cue to switch.
- A *response network* that selects an association as a response based on the current primary cue and previous responses.

While all three cues are being provided as input to the model, only one of them at a time will be regarded as the primary cue to generate associations. This is consistent with the way humans generate responses (Smith et al., 2013). To select a single cue each input cue is fed through a group of gating neurons that project to the neurons representing the primary cue. Inhibiting a set of gating neurons will prevent the transmission of the corresponding cue. To select a single cue, a simple winner-take-all (WTA) mechanism, seeded by white noise, is used. To get the required inhibitory gating signal, the WTA output has to be inverted. This is done with additional groups of neurons biased to represent 1. The WTA output will inhibit one of these groups to deactivate its inhibition of the gating neurons.

In the response network a single associated word is selected by a clean-up memory (Stewart et al., 2011b) with an added WTA mechanism. The input vector is correlated with the individual word vectors and each correlation value is represented by a group of neurons. In this way, the preferred direction vectors are not randomly distributed as in other parts of the model, but the preferred direction of every neuron and every group of neurons corresponds to one of the words. Furthermore, these groups of neurons threshold the represented value at 0.1. Each group is connected with every other group with lateral inhibitory connections, and to itself with a self-excitatory connection. This allows the group with the strongest input to remain active, while inhibiting all other groups. Another feedback connection is used to capture the evidence on the locality of search (Smith et al., 2013). This connection implements a transformation $\tilde{A}$, so that the associates of the current response are fed as additional input to the WTA network. In Figure 2, all of these recurrent connections are denoted by a single feedback connection on the WTA in the response network.

The response inhibition plays a crucial role in allowing the next word to appear in the search process. It is realized as a neural group acting as a leaky integrator. Without external input, the represented vector will slowly decay to the zero vector. A recurrent connection feeding the output of the neural group back to itself prevents the otherwise very quick decay. External input to the integrator will slowly shift the represented value toward the input vector. This input is provided from the WTA network, while at the same time the response inhibition is used to inhibit the WTA network. Thus, the active word in the WTA network will be subject to increasing inhibition until finally a new word is selected. This switch will typically happen before the vector represented by the response inhibition shifted completely to the input vector. Because of that, the response inhibition will represent an additive mixture of the sequence of the last couple words and prevents those from reappearing in the search process in short succession.

The list of free model parameters and their values which produce the described model behavior is provided in Table 1. The values have been determined manually by observing which ranges produce the desired word-selection behavior.

**Table 1:** **Free parameters of the RAT model**.

**Parameter**	**Value**	**Description**
d	2048	Number of dimensions per word vector
th	0.6–0.8	Percentage of randomly removed associations in the association matrix (varies with simulation)
assoc_th	0.05	WTA cut-off input threshold
cue_strength	0.1	Input strength of individual cues to WTA network
primary_cue_strength	0.7	Input strength of primary cue to WTA network
wta_feedback_strength	0.5	Input strength of associates of current response to WTA network
noise_std	0.01	Standard deviation of the zero-centered Gaussian noise in the cue selection network
integrator_feedback	0.95	Strength of recurrent connection on response inhibition

To generate the association transformation matrix $\tilde{A}$, we used the Free Association Norms dataset (FAN; Nelson et al., 2004). This dataset was constructed from a series of free association experiments conducted with over 6000 participants over the course of a few decades. In the experiments, participants were presented with a cue word and asked to write down the first word they thought of. In this way, a distribution of associates was created for every cue word by norming the frequency of response with the number of participants performing the task. The FAN data have been shown to provide a good match with human performance on the RAT when using it for solving RAT problems with a 2 s time limit (Kajić et al., 2016). Here, we use a binary association matrix ***A*** by assigning 1 to all non-zero association strengths and 0 otherwise. This disregards the associative strengths and only considers the (non-) existence of associative links. Preliminary simulations indicated that this approach gives a better match to the human data than weights proportional to the frequency of associations. To model individual differences in associative networks and adjust solution probabilities to match human data, we randomly remove between 60% and 80% of associations in the matrix by setting them to zero. This range has been determined empirically.

Not all potential responses produced by the response network qualify as a valid response to a RAT problem. Some words might be the result of an implicit priming effect, where a previous response primed a word which is not related to any of the cues. Also, it is reasonable to assume that participants in the experiment have typed only a subset of words that they thought of. To account for these effects, we implement a filtering procedure that regards only certain words as responses to a RAT problem. For every generated word, a similarity measure to the problem cues is calculated and, if it is below a threshold, the word is dismissed. The similarity is the sum of association strengths between every cue and the word.

Prior work (Kajić et al., 2016) allowed us to focus on a single source of association data for the generation of potential responses. However, we have no reason to assume that the same data is optimal for the filtering procedure. As such, we explore two association matrices and their binary variants for filtering purposes: FAN and the Google Books Ngram Viewer dataset (version 2 from July 2012, Michel et al., 2011, here referred to as Ngrams). We have previously shown both datasets to be suitable for modeling the RAT (Kajić et al., 2016). Although, the two sources of data contain similar information, there are interesting differences: approximately 6.5 million association pairs exist in the Ngram matrix and not in the FAN matrix. Conversely, only about 26,000 associations exist in the FAN but do not exist in the Ngram matrix.

Unlike the Ngram matrix, the FAN matrix contains non-reciprocal association strengths because associations are not bi-directional. Ninety-four percent participants in the free association experiment responded with the word *right* when given a cue *left*. However, the cue word *right* has much lower association to the word *left*, as only 41% participants responded with *left* and 39% participants responded with *wrong*. We used the sum of the FAN matrix with its transpose to obtain a symmetric association matrix.

While FAN data provides empirically derived association information through experiments with humans, a co-occurrence matrix for the Ngram data set has been derived by counting frequencies of n-grams across 5 million books published up to 2008. This is the second matrix we use. Here, we focus on 2-gram (bi-grams) only for words which exist in the FAN database. The Ngram matrix was constructed by iterating over all combinations of associative word pairs *w*_1_ and *w*_2_ and summing up occurrences of the 2-gram (*w*_1_, *w*_2_) and the 1-gram *w*_1_*w*_2_ in the corpus.

Apart from using matrices that contain association strengths (for FAN) and co-occurrence frequencies (for Ngrams), we also explore whether just the existence of an association is sufficient to obtain the distribution of responses similar to the distribution of human responses. This is easily achieved by setting every non-zero entry in the matrix to one and gives the binary matrices bFAN and bNgram.

### 2.4. Model evaluation

To evaluate the model, we use a set of 25 RAT problems and compare the model responses to the human responses from Smith et al. (2013). For each of the 25 problems, we ran 56 simulations with different random number seeds to ensure the independence of the results from the initial conditions, such as the choice of neurons and word vectors. For the analysis of responses, we adapt a set of analysis tools from Smith et al. (2013), which was originally developed to analyze human responses and characterize memory search in the RAT. The same analysis tools are used for human responses and model responses. While the experimental details about the data collection and detailed descriptions of analysis methods are available in the original publication, we present a brief overview of the data and a description of the adapted methods.

The data set contains responses from 56 participants, which were given 2 min to solve each RAT problem. Every participant was given 25 problems and was instructed to type every word which came to their mind, as they were solving the problem. Participants indicated when they thought they had provided the correct solution word with a key press. Thus, every trial consists of a sequence of responses from one participant to one RAT problem, ideally ending with the correct solution. Here, the analysis of responses has been performed over 1396 human trials and 1400 model trials. For each RAT problem, we ran 56 simulations, corresponding to the number of human participants. In 169 trials, human participants marked an incorrect response as correct and we excluded those from qualitative analyses, as they could have skewed analyses comparing how participants approached the final answer on incorrect and correct trials.

For every trial we did a series of pre-processing steps, as per Smith et al. (2013). Word pairs with words not available in the Free Norms or words identical to one of the cues were excluded from the analysis. Responses repeated twice in a row were merged into a single response. Then, we assigned a 300-dimensional word vector to every word, including problem cues, the solution, and human responses. Those vectors were based on the Word Association Space (WAS; Steyvers et al., 2004), constructed by reducing the dimensionality of an association matrix. This matrix was the WAS ***S***^(2)^ measure based on the FAN, which includes not only direct association strengths between two words ***w***_*i*_ and ***w***_*j*_, but also links across one intermediary word, i.e., associations from ***w***_*i*_ to ***w***_*k*_ to ***w***_*j*_. The similarity between words was measured as the cosine angle between the assigned word vectors. To conclude the pre-processing, every response was assigned the word vector with the highest similarity as the primary cue vector.

Metrics were calculated on the pre-processed data to evaluate the model. First, we determined the *average response similarity* for within and across cluster response pairs of adjacent responses. Clusters were defined on the primary cue of the responses; adjacent responses with the same primary cue are considered to be part of the same cluster. This was done to test for bunching of responses around cues by comparing the similarity between word pairs in each cluster. The assumption is validated with a *permutation test for average response similarity* by assigning cues from another trial and checking for conservation of similarity trends. The average response similarity within clusters is also computed in a cleaned data set, where all missing entries were dropped, which yielded new response pairs. Second, the *probability of switching primary cues* is computed as the number of response pairs with the different cues divided by the total number of response pairs. This value needs to be compared against a baseline probability based on the frequency each cue was selected under an independence assumption. This baseline calculation is required because certain cues might be selected more or less often than pure chance would predict. Third, the *similarity between adjacent and non-adjacent responses* within a cluster is computed to test for the direct influence of the previous response on the next one. The same is done for the responses with different primary cues, which occur right at the cluster breaks. Fourth, we tested whether the similarity to the final response increases as participants approach the final answer (either correct or incorrect).

## 3. Results

In this section model responses are presented and compared to human responses using the methods described. Quantitative comparisons refer to the statistics of responses in terms of the number of correct solutions and the average number of responses for each RAT problem. The qualitative analysis addresses semantic properties of responses. Semantic analysis is based on the WAS space as described in Section 2.4. The aim of the qualitative analysis is to investigate whether response search trends, observed in human responses, match with those produced by the model. In particular, this refers to bunching of responses around problem cues, local search strategy, and clustering patterns.

### 3.1. Quantitative comparison

**Figure 3:**
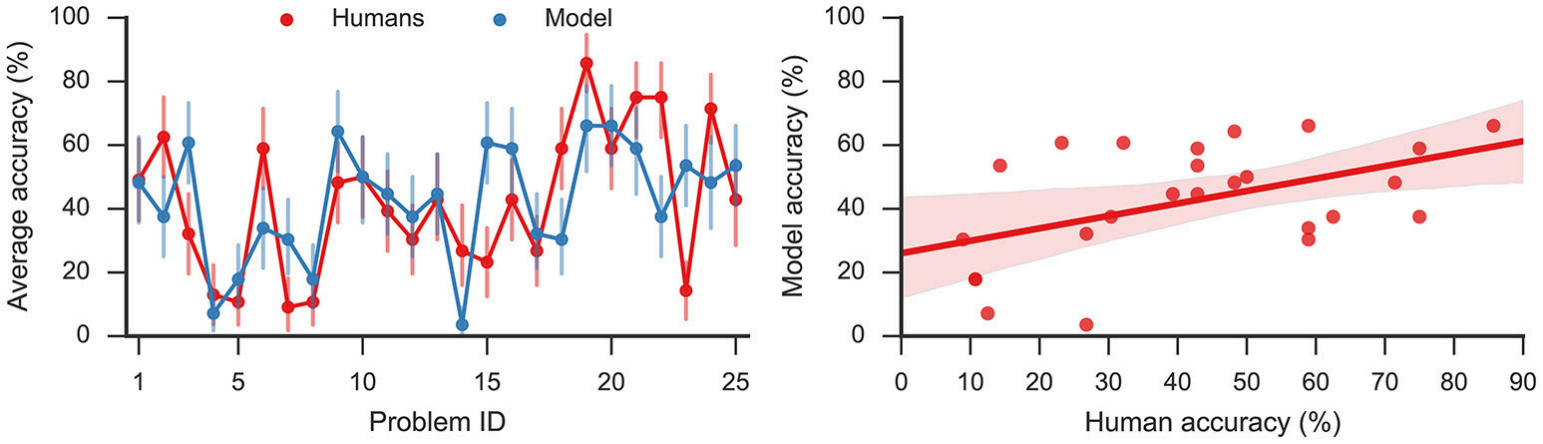
**(Left):** Average accuracy on 25 RAT problems for model responses and human responses. Error bars denote 95% bootstrap confidence intervals. **(Right):** Linear regression (Pearson correlation coefficient *r* = 0.49, *p* < 0.05) with 95% bootstrap confidence intervals.

The model solved on average 43% of the problems, showing a moderate correlation (Pearson correlation coefficient *r* = 0.49, *p* < 0.05) with humans who on average solved 42% problems. The left panel of Figure 3 shows the accuracy on the 25 problems averaged, respectively, over all model simulations and over all human subjects. By applying the two-sided exact binomial test we find that for 14 out of 25 problems there is a statistically significant difference (*p* < 0.05) between the human and model responses^3^. These results are expected given that there are some problems which are easier for humans, and others that are easier for the model. On two problems—*dust, cereal, fish*; and *speak, money, street*—the model accuracy was more than 35 percentage points greater than the human accuracy on the same problems. On the other hand, there was one problem, *safety, cushion, point* where the human score was more than 35% points higher than the model score. However, Table 2 indicates that, while the accuracy of this model matches well to the human performance, this model produces a much longer sequence of outputs than observed in the human subjects (40.20 vs. 7.78).

**Table 2:** **Quantitative analysis of raw and filtered model responses**.

**Analysis**	**Humans**	**Raw**	**Filtering method**			
			**FAN**	**bFAN**	**Ngram**	**bNgram**
Filtering threshold			0.006	1	0.006	3
Shortest response sequence	1	2	1	1	1	1
Longest response sequence	49	46	39	40	27	33
Mean response sequence length	7.78	40.20	16.99	17.47	8.33	8.44
- Correlation with human data (*r*)		−0.30^*^	0.54^***^	0.51^***^	0.95^***^	0.93^***^

Association matrices used for the filtering are: FAN, Free Association Norms; bFAN, binary FAN; Ngram; bNgram, binary Ngram. Values significant at p < 0.05 are marked with ^*^, significant at p < 0.001 with ^***^.

To deal with this discrepancy, we consider that there is some filter applied between the output of the model and the actual reported responses (in other words, the subjects do not actually write down all the words that come to mind while performing the task). As described in Section 2.3, this means that only a subset of all words produced by the model will be regarded as a set of responses to a RAT problem. In particular, a word that has a connection strength to all three cues below a threshold will be discarded. Thresholds have been determined as the lowest connection strength between the sets of three cues and solution for all problems. In this way, filtering will ensure that all solution words pass the filter. As a result, the accuracy and the correlation with the human accuracies are independent of the filtering method. Table 2 summarizes the statistics for the raw data and various filtering methods. We compared the average number of responses per trial, the shortest and longest response sequence, and the match between distributions of the number of responses.

**Figure 4:**
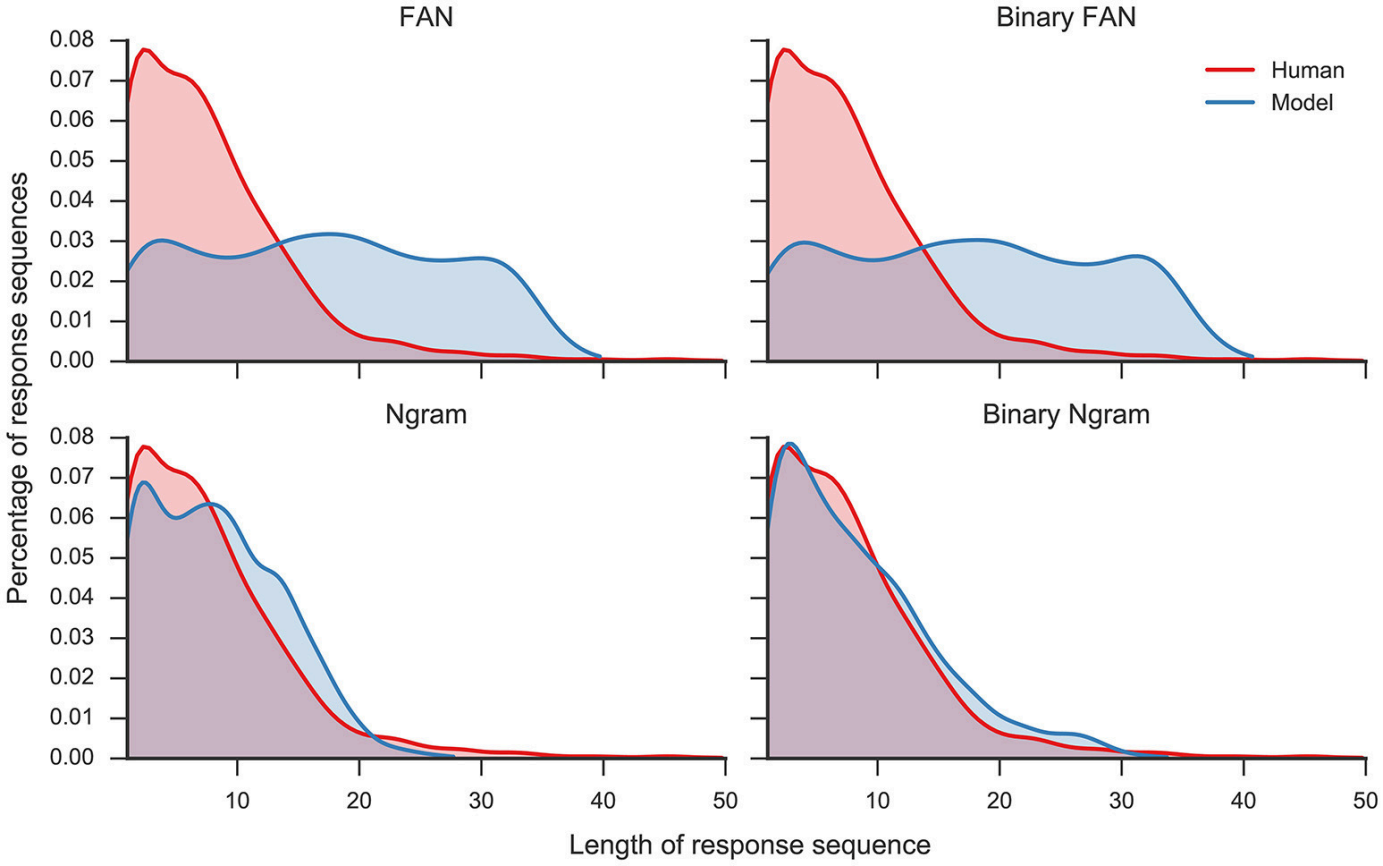
**Distribution of number of responses per trial for human responses and model responses plotted as Kernel Density Estimates (Gaussian kernel, bandwidth determined with Scott's rule, Scott, 1979)**. Four different model distributions were produced with four different filters (see text for details): Free Association Norms (FAN), binary FAN, Ngram, and binary Ngram.

Overall, the Ngram matrix and the binary Ngram matrix yield distributions that best match to human data (*r* = 0.95 and *r* = 0.93, respectively). The threshold for the binary Ngram matrix has been set to 3, so that a word will pass the filter if it is an associate of all three problem cues. Reducing the threshold to two decreases the correlation with the distribution to *r* = 0.36 (*p* < 0.05) and increases the average number of responses per problem to 17.73. Figure 4 displays the distributions for all filters plotted against the distribution of human responses. The Ngram derived matrices are more aggressive in filtering the responses compared to the FAN derived matrices. The former preserve ≈ 20% of words produced by the model, while the latter did so for ≈ 40% of the responses. Although, the Ngram matrix and the binary Ngram matrix yield comparably good matches with response distributions, in the following analyses we use the binary Ngram matrix which provided a slightly better match for some of the qualitative analyses.

To further investigate the effects of this filter, we tried applying it to the human data. Similarly to the model data it also reduced the dataset considerably, leaving <10% of overall responses.

### 3.2. Qualitative comparison

We analyze responses obtained by applying the filter to raw model outputs in terms of their semantic similarity. The analysis compares similarity between two groups of response pairs, where groups refer to primary cue assignment of response pairs (same cue vs. different cue) and their proximity in a sequence of responses (adjacent vs. non-adjacent word pairs). Such analyses on human responses (Smith et al., 2013; Davelaar, 2015) showed that responses humans give tend to bunch around one of the three problem cues, and that different cues can be selected while search for the solution unfolds. Also, responses show sequential dependence, where the next response is dependent on the previous one. We use the set of analysis methods described in Section 2.4 to explore whether model responses exhibit such similarity patterns.

All analysis results are summarized in Table 3. To test for bunching of responses around problem cues, we explore the similarity of response pairs with a common primary cue. The similarity is greater for word pairs with the same cue compared to word pairs with different cues [0.141 vs. 0.054; two-sided *t*-test *t*_(9915)_ = 20.4]. This trend is preserved when we use the permutation test, which randomly assigns cues from a different trial [0.142 vs. 0.054; *t*_(4729)_ = 13.7]. Evidence for sequential dependence of word responses has been found by comparing similarities for word pairs within the same cluster; adjacent word pairs within the same cluster are more similar than pairs which are further apart [0.141 vs. 0.076; *t*_(13652)_ = 17.8]. Additional evidence for sequential search arises from greater similarity between adjacent word pairs with different primary cues compared to non-adjacent word pairs with different primary cues [0.054 vs. 0.011; *t*_(12,819)_ = 22.4]. We found that when the model produced a response, it produced another response with the same primary cue in 54.4% of cases. As done in the previous studies (Smith et al., 2013; Bourgin et al., 2014), we also analyzed the change in similarity between the final response (either correct or incorrect) and each one of the ten words prior to the final response. We identified a positive slope in similarity rates as responses were approaching the final answer.

**Table 3:** **Performance on the RAT and similarity patterns in the response search**.

**Analysis**	**Humans**	**Model**
Average problem accuracy	42%	43%
-Correlation with human data (*r*)		0.49^*^
Shortest response sequence	1	1
Longest response sequence	49	33
Average number of responses per trial	7.78	8.44
-Correlation with human data (*r*)		0.93^***^
**AVERAGE RESPONSE SIMILARITY**		
-Within vs. across cue clusters	0.189 vs. 0.041	0.141 vs. 0.054
	CI: [0.134, 0.162]	CI: [0.079, 0.095]
-Permutation test	0.182 vs. 0.040	0.142 vs. 0.054
	CI: [0.124, 0.160]	CI: [0.077, 0.100]
-Within vs. across cue clusters (cleaned responses)	0.180 vs. 0.039	0.141 vs. 0.054
	CI: [0.128, 0.154]	CI: [0.079, 0.095]
Baseline vs. actual percentage of response pairs with the same primary cue (two-sided exact binomial test)	33.3 vs. 37.1%^***^	34.2 vs. 54.4%^***^
**AVERAGE SIMILARITY BETWEEN ADJACENT AND NON-ADJACENT**		
**RESPONSES**		
-With different primary cues (across cluster)	0.041 vs. 0.016	0.054 vs. 0.011
	CI: [0.063, 0.098]	CI: [0.038, 0.047]
-With same primary cues (within cluster)	0.189 vs. 0.108	0.141 vs. 0.076
	CI: [0.063, 0.098]	CI: [0.057, 0.072]

Stated 95% confidence intervals are computed on the difference of reported mean values. Values significant at p < 0.05 are marked with ^*^, significant at p < 0.001 with ^***^.

### 3.3. Neural outputs

We now turn to the neural responses generated by the model. Consequently, most observations in this section can be regarded as qualitative comparisons to spiking patterns observed in cortical neurons.

**Figure 5:**
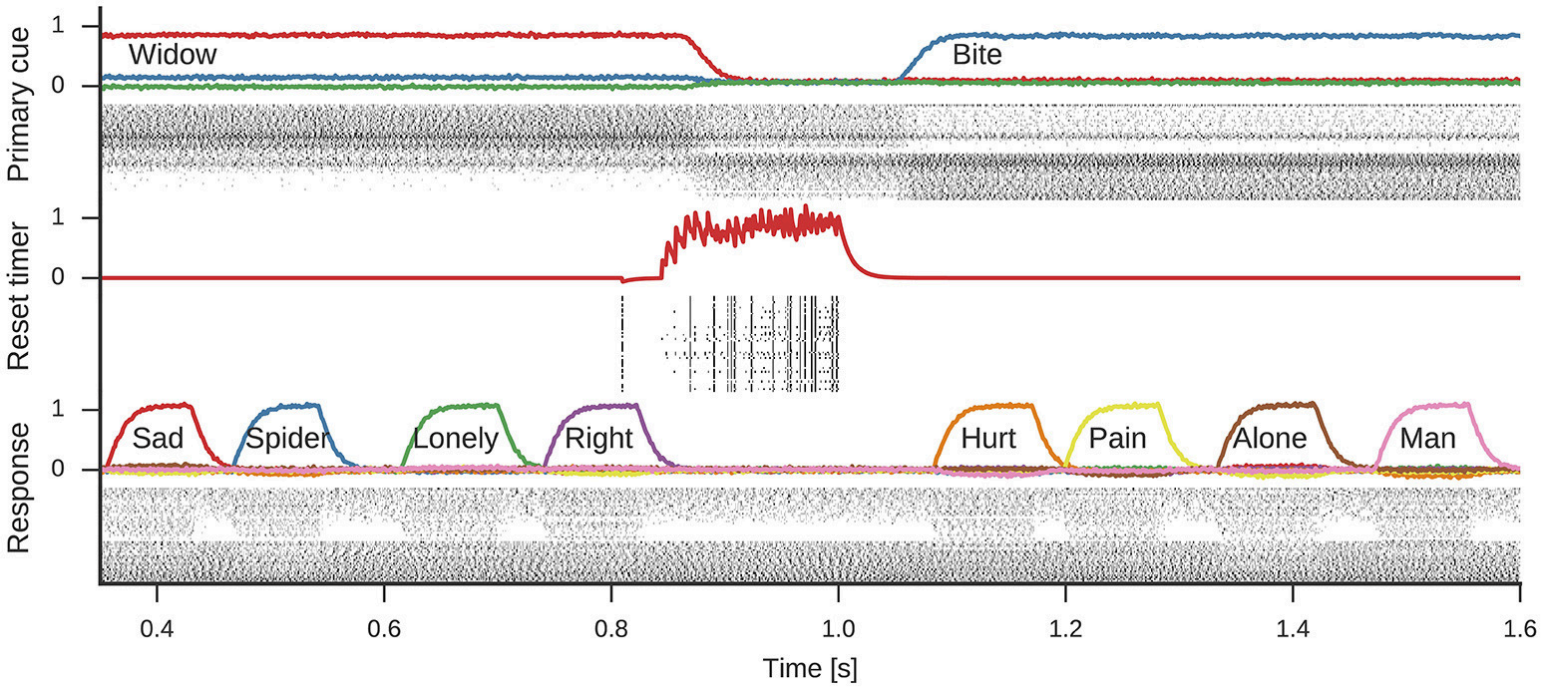
**Spikes and decoded values for three neural groups in the model**. Data shown are an excerpt from a longer single simulation run. From top to bottom data for neurons representing the *primary cue*, the cue selection *reset signal*, and the *response* neurons are shown. Line plots for the primary cue and response show the similarity of the decoded vector with word vectors and are annotated with the corresponding words. The reset signal line plot shows the decoded scalar value. These line plots are interleaved with corresponding spike raster plots showing a subset of the neurons partaking in the representations.

Figure 5 shows the spiking activity in three parts of the model during one simulation run. In the shown time frame, the primary cue starts as *widow*, but changes to *bite* about halfway through. This change is induced by the rising reset signal inhibiting the cue selection and causing a reselection of the primary cue. During the active period of either cue, the response neurons sequentially represent different words associated to the cue. Note, while four associations are shown for either cue, the number of responses generated during each active phase of a primary cue differs.

**Figure 6:**
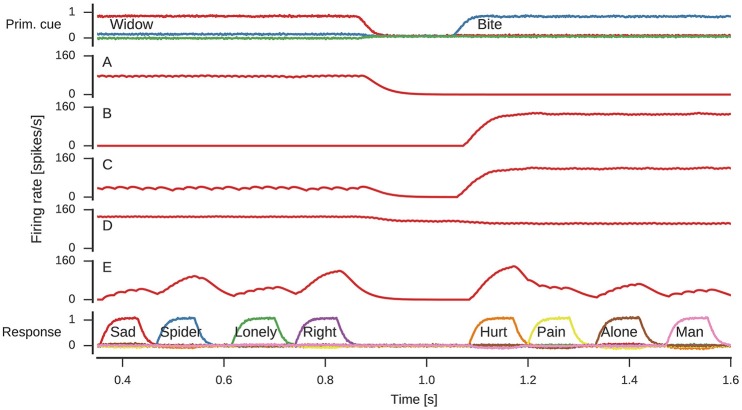
**Firing rates of individual neurons**. Spike trains where filtered with $h\left(t\right)={\left[\alpha^{2}t\exp\left(-\alpha t\right)\right]}_{+}$ to obtain firing rate estimates. **(A)** Neuron responding to *widow*. **(B)** Neuron responding to *bite*. **(C)** Neuron responding to both *widow* and *bite* to a varying degree. **(D)** Neuron responding to both *widow* and *bite* with a more subtle difference. **(E)** Neuron responding to varying degrees whenever a response is produced.

The spike raster plots (Figure 5) and firing rate estimates in Figure 6 reveal interesting neuron tuning properties. We observe neurons that appear to be selective to cue words: some neurons only fire for *widow* (Figure 6A), while others only fire for *bite* (Figure 6B) in the shown time span. However, it is important to note that we did not test the response of these neurons to all possible cues and there might be other words which also elicit their response. Notwithstanding, such selective and explicit response behavior is consistent with observations from single-neuron recordings in medial temporal cortex in humans (Földiák, 2009; Quian Quiroga, 2012). We also observe neurons that fire for both cues, but with different firing rates. This word-dependent change in firing rate is more prominent for some neurons (Figure 6C), while it is more subtle for others (Figure 6D). The response population also includes neurons that are primarily active when a word is being represented, but not otherwise (Figure 6E).

From a single neuron perspective, of particular interest is the reset signal. Here, the neurons produce a clear bursting pattern during the onset of the reset signal. Such behavior is often thought to need an explanation in terms of complex neuron models that intrinsically burst (Izhikevich, 2007), which is not a charactersitic of LIF neurons. Nevertheless, we observe a bursting behavior because of the recurrent network dynamics producing the reset signal.

The presented neural network model, constrained by biological properties like membrane and synaptic time constants, shows a reasonable match to behavioral data. With that in mind, we believe that proposed mechanisms, such as word selection and word inhibition realized in spiking neurons, demonstrate the biological plausibility of this approach. Future work can address a stronger claim about the connection between measurable neural signals and the proposed mechanisms by using the model to generate fMRI predictions from the physiological properties of spiking neurons and their dendritic activity, as done in previous work (Eliasmith, 2013, Chapter 5).

## 4. Discussion

We proposed a spiking neural network model that solves the Remote Associate Test, a task commonly used in creativity research. The model shows a significant correlation with human accuracy on the test, and its responses replicated similarity patterns observed in human responses (Smith et al., 2013; Davelaar, 2015). At the same time it implements possible biological mechanisms that generate these behavioral patterns, thus connecting multiple scales of the cognitive system.

The existing body of modeling studies have contributed to the general understanding of the RAT, including factors that influence the difficulty. Specifically, word frequency has been investigated as important in determining solvability of a RAT problem; an aspect that was already discussed when the test was developed (Mednick, 1962). Based on the frequency of a word or an expression in text corpora, it is possible to determine whether a problem will be easy or hard for humans (Gupta et al., 2012; Olteteanu and Falomir, 2015). Our model has reproduced the pattern of RAT item difficulty by showing a correlation with human accuracies on the 25 problems from Smith et al. (2013). Individual differences in associative networks known to influence the performance on the test (Kenett et al., 2014) were modeled by randomly dropping a fraction of associations from the association matrix. Moving beyond the accuracy measure, we also looked at the quantitative and qualitative characteristics of response sequences. In terms of quantitative statistics, we analyzed the distribution of response sequence lengths which showed a strong correlation with the human data. We also observed a good match of the model and human data with respect to qualitative properties, such as bunching of responses around a single cue, cue switching, and sequential search. Such statistical similarity patterns were also successfully reproduced with probabilistic approaches in Bourgin et al. (2014), but without reference to cognitive processes underlying the search. Our model extends current findings by proposing biologically plausible network components involved in the search. In addition, we demonstrated how the representations in the model can display both specificity (commonly attributed to localist representation) and broad tuning (commonly attributed to distributed representation) depending on how single neuron activity is analyzed.

Previous studies identified the FAN as a viable source of associative data to model the RAT (Gupta et al., 2012; Bourgin et al., 2014; Kajić et al., 2016) and provided the motivation to use it in this model. Steyvers and Tenenbaum (2005) have shown that the FAN exhibits small-world properties. That means that its shortest paths between nodes are short on average, the clustering coefficients are high, and the connectivity is sparse. In our model, however, we removed associative links to model individual differences. It is left to future research to explore how this changes the properties of the associative network. For example, it might be possible that the small-world property gets disrupted leading to a lower performance on the RAT (Kenett et al., 2014).

Besides the generation of potential responses, we identified that it is important to filter out some of these responses to match human data. Interestingly, the Ngram data proved to be better suited for this task than the FAN. This leads to the hypothesis that humans use both sorts of information at different stages in the search process. But the cause could also be that most solutions, in the set of 25 problems, created compound words or word phrases with the cues, which is a property reflected to a larger degree in the co-occurrence data of the Ngrams. Nevertheless, it remains interesting that the Ngram data does not seem to be used for the generation of potential responses (Kajić et al., 2016).

While the current model offers a first unified account of the RAT search process in terms of both psychological and biological mechanisms, significant possible improvements remain for future work. First, switching of the primary cue is induced in quite regular intervals in our model. While we cannot exclude the possibility that this is the case in the actual cognitive process, we expect the actual process to be more complex. It would be interesting to explore how changing this part of the model can improve the match to human data, especially regarding the percentage of response pairs with the same primary cue. Second, the filtering of potential responses could be further investigated by exploring methods which discard less of the human and model responses, providing a closer match with the plausible cognitive mechanism. Furthermore, biologically plausible filtering with neurons should be implemented to extend the plausibility of the mechanisms of the complete model. While we have a proof-of-concept implementation of the filtering methods in spiking neurons, it is not yet complete. Third, current analysis methods filter out repeated responses, but these might give additional information on the search process and considering their occurrence patterns would allow us to refine the response inhibition network. Finally, the current model does not explain how humans learn word associations, or how the process of learning relates to changes in connection weights that store the relevant information. Since the acquisition of linguistic structure happens early in childhood and continues to develop throughout adulthood (Elman et al., 1997), a full account of word representation in the brain would also need to address learning at multiple time-scales, as well as mechanisms which enable such learning.

## 5. Conclusion

The RAT model proposed here specifies both cognitive processes and their neural implementation, which makes it unique among models of the RAT task. The model was validated on empirical data and shows a good match to this data. In the process of matching this data we identified that two processes might be at work: the generation of potential answers and the filtering of the answers to provide reported responses. Furthermore, the model sheds light on how the task relevant information can be represented in biologically realistic spiking neurons.

## Author contributions

Conceived the model and experiments: IK, JG, TS, TW, and CE. Implemented the model: IK, JG. Analyzed the data: IK, JG. Wrote the paper: IK, JG, TS.

## Funding

This work has been supported by the Marie Curie Initial Training Network FP7-PEOPLE-2013-ITN (CogNovo, grant number 604764), the Canada Research Chairs program, the NSERC Discovery grant 261453, Air Force Office of Scientific Research grant FA8655–13–1–3084, CFI, and OIT.

### Conflict of interest statement

The authors declare that the research was conducted in the absence of any commercial or financial relationships that could be construed as a potential conflict of interest.

## Footnotes

^1^This assumes no synaptic weight changes, e.g., through learning, are happening. These could also be handled in a biologically realistic manner by the NEF (Bekolay et al., 2013), but are out of the scope of this paper.

^2^Semantic Pointers for words are more sophisticated than the representations used here, as they can encode structured representations as well. However, those structured representations are also vectors encoded as described here, so the present model is consistent with using semantic pointers for cognitive representation more generally.

^3^It should be noted that if the number of problems is increased sufficiently, then there will always be a statistically significant difference for all conditions. For this reason, we take this test as a means of identifying problems where model responses deviate the most from human responses rather than as a measure of the quality of the model.
