# Supplementary material for: A Spiking Neuron Model of Word Associations for the Remote Associates Test
Source: Front Psychol. 2017 Feb 2;8:99. doi: 10.3389/fpsyg.2017.00099 (PMC5288385; doi:10.3389/fpsyg.2017.00099)
Supplement: Supplementary file 1 [file DataSheet1.PDF]

Supplementary Material: A Spiking Neuron Model of Word Associations for the Remote Associates Test

Ivana Kajić*, Jan Gosmann, Terrence C. Stewart, Thomas Wennekers and Chris Eliasmith

*Correspondence:
Ivana Kajić
i2kajic@uwaterloo.ca

1 MODEL DETAILS

This material provides technical and implementational model details which were omitted in the main text. First, we introduce general building blocks and terminology, followed by an explanation of how these are used to construct parts of the model.

1.1 Building blocks

The most basic building block in the context of the Neural Engineering Framework (NEF; Eliasmith, 2013) is a group of neurons used to represent d-dimensional vectors. We call such a group of neurons a neural *ensemble*. While in theory it is possible to represent vectors of any dimensionality in a single ensemble, this has some practical problems. The representation requires a large number of neurons and the least-square optimization for the decoding weights will take a very long time. Instead we split the 2048-dimensional vectors into smaller vectors of 16 dimensions and use one ensemble for each 16-dimensional part. This yields 128 ensembles per vector representation.

We use 50 neurons per represented dimension in each ensemble, that is 50 neurons in ensembles representing a scalar and 800 neurons in ensembles representing 16-dimensional vector parts. Connections between ensembles use exponential $h(t) = \frac{1}{\tau}\exp(-t/\tau)$ synapses with a time constant of $\tau = 5\,\mathrm{ms}$ if not otherwise noted. Further model parameters are given in Section 1.3.

1.1.1 Thresholding

In several places in the model scalar values are thresholded. The preferred directions of all neurons in the corresponding ensembles are set to 1, thus all the neurons increase firing for larger represented values. Furthermore, we choose the tuning curves of the neurons in a way that no neuron fires below the threshold value and most neurons will start firing for values near the threshold.

1.1.2 Winner-Take-All

A winner-take-all (WTA) network to chose from n options is implemented with n ensembles. The ensembles are configured to support thresholding and have lateral inhibitory connections. In this way every group of neurons projects the negative of its represented value to all other groups.

1.1.3 Gating neurons

The gating neurons in the model are regular ensembles representing 16-dimensional vector parts. They receive an additional inhibitory input that suppresses the firing of the neurons. With all firing suppressed, the null vector is implicitly represented. Thus, the inhibitory input allows to determine whether a vector is transmitted to the post-synaptic neurons.

1.1.4 Integrators

The activity of a group of LIF neurons will quickly decay without external input. By adding a recurrent connection the neurons can drive themselves and store a value over an extended time frame. Additional external input will be added to the stored value which makes such a group of neurons an integrator.

1.2 Model networks

We use these basic building blocks to construct more complex networks that constitute our model. Here, we describe the reset signal and response inhibition that have not been described in the main text.

1.2.1 Reset signal

Figure S1 shows the details of the network providing the reset signal for the cue selection. To produce the signal, the *ramp* ensemble integrates a constant bias signal of 0.1. The synaptic time constant on the recurrent connection of this ensemble is set to $0.1\,\mathrm{s}$. The *ramp* output is fed to the *switch* thresholding ensemble with a set threshold of 0.9. When the threshold is reached, it triggers the inhibition of all neurons in the cue selection WTA network. This restarts the cue selection process.

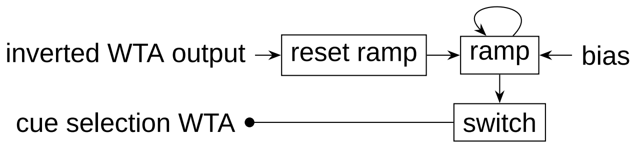

Figure S1. The reset signal network.

Restarting the cue selection process also requires resetting the *ramp* signal. Note that while usually only two of the inverting ensembles after the WTA will represent 1 (inhibiting the transmission of the two non-primary cues), with the inhibition of the WTA by the reset signal all three will represent 1 (inhibiting the transmission of all three cues as none is selected as primary cue). The sum of these three ensembles divided by 3 is fed to another thresholding ensemble *reset ramp* with a threshold of 0.9 which thus will only be exceeded if no primary cue is selected. This connection uses a slow time constant of $0.1\,\mathrm{s}$ to ensure a sufficiently long signal. The *reset ramp* signal is then multiplied by -5 and fed into the *ramp* neurons to reset its value.

1.2.2 Response Inhibition

The response inhibition (Figure S2) consists of an integrator group of neurons. The recurrent connection uses a time constant of $0.1\,\mathrm{s}$ and is adjusted to have a strength of 0.95, so that the stored vector slowly decays. In this way this integrator stores a vector which is an additive combination of recent responses. The output is correlated with all word vectors and fed to another set of thresholding neurons (threshold 0) providing inhibition to the response WTA to suppress recent responses.

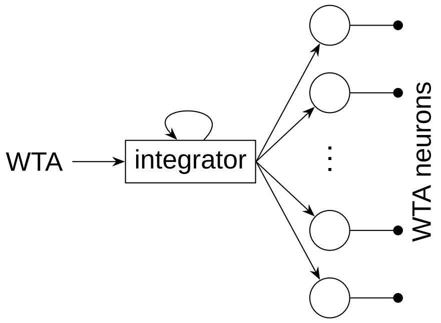

Figure S2. Response inhibition network.

1.3 Model parameters

Parameters used in the model if no other value was given in the text.

Parameter	Value
LIF neuron model	
– Membrane time constant	$20\,\mathrm{ms}$
– Refractory period	$2\,\mathrm{ms}$
Synapses	Exponential with $\tau = 5\,\mathrm{ms}$
Ensembles	
– Maximum firing rates (over all vectors of length 1)	Random uniform distribution between $200\,\mathrm{Hz}$ to $400\,\mathrm{Hz}$
– Neurons per represented dimension	50

REFERENCES

Eliasmith, C. (2013). *How to build a brain: A neural architecture for biological cognition* (New York, NY: Oxford University Press)
